# Lipidomics reveals accumulation of the oxidized cholesterol in erythrocytes of heart failure patients

**DOI:** 10.1016/j.redox.2017.10.020

**Published:** 2017-10-26

**Authors:** Hsiang-Yu Tang, Chao-Hung Wang, Hung-Yao Ho, Pei-Ting Wu, Chun-Ling Hung, Cheng-Yu Huang, Pei-Ru Wu, Yung-Hsin Yeh, Mei-Ling Cheng

**Affiliations:** a Metabolomics Core Laboratory, Healthy Aging Research Center, Chang Gung University, Taoyuan Taiwan; b Heart Failure Research Center, Division of Cardiology, Department of Internal Medicine, Chang Gung Memorial Hospital, Keelung, Taiwan; c College of Medicine, Chang Gung University, Taoyuan, Taiwan; d Department of Medical Biotechnology and Laboratory Science, College of Medicine, Chang Gung University, Taoyuan, Taiwan; e Clinical Phenome Center, Chang Gung Memorial Hospital, Taoyuan, Taiwan; f Graduate Institute of Biomedical Sciences, College of Medicine, Chang Gung University, Taoyuan, Taiwan; g Department of Biomedical Sciences, College of Medicine, Chang Gung University, Taoyuan, Taiwan; h Cardiovascular Division, Chang-Gung Memorial Hospital, Chang-Gung University College of Medicine, Chang-Gung University, Taiwan

**Keywords:** Heart failure, Lipidomics, 7-ketocholesterol, Oxidative stress

## Abstract

Lipids play an important role in the pathogenesis of cardiovascular disease. Changes in lipids of erythrocytes are indicative of the outcome of pathophysiological processes. In the present study, we assessed whether the lipid profiles of erythrocytes from heart failure (HF) patients are informative of their disease risk. The lipidomes of erythrocytes from 10 control subjects and 29 patients at different HF stages were analyzed using liquid chromatography time-of-flight mass spectrometry. The lipid composition of erythrocytes obtained from HF patients was significantly different from that of normal controls. The levels of phosphatidylcholines (PCs), phosphatidylethanolamines (PEs), and sphingomyelins decreased in HF erythrocytes as compared with those of control subjects; however, the levels of lysoPCs, lysoPEs, and ceramides increased in HF erythrocytes. Notably, the oxidized cholesterol 7-ketocholesterol (7KCh) accumulated to higher level in HF erythrocytes than in plasma from the same patients. We further validated our findings with a cohort of 115 subjects of control subjects (n=28) and patients (n=87). Mechanistically, 7KCh promoted reactive oxygen species (ROS) formation in cardiomyocytes; and induced their death, probably through an ATF4-dependent pathway. Our findings suggest that erythrocytic 7KCh can be a risk factor for HF, and is probably implicated in its pathophysiology.

## 1 Introduction

Cardiovascular disease is a major health problem and the leading cause of death globally. Cardiac function deterioration hampers the ability of the heart to support blood circulation, resulting in heart failure (HF). The pathogenic mechanism leading to this end stage is complicated. Myocardial infarction, hypertension, cardiomyopathy, valvular heart disease, and inflammation-induced oxidative stress are known risk factors for disease progression [1], [2].

Several biochemical pathways, including the pentose phosphate pathway, anaplerotic metabolism, ketone body metabolism, lipotoxic intermediate metabolism, and glycolysis, are affected in patients with HF [3], [4], [5]. Changes in metabolites have been identified in plasma and are associated with clinical outcomes in patients with HF [6], [7]. These findings suggest that metabolic remodeling in patients may occur during HF progression, and the metabolite profile can thus be used as a biomarker panel for a variety of assessment purposes.

Lipid metabolism alterations have been increasingly demonstrated to underlie the pathogenesis of cardiovascular disease [8]. Currently, research on lipids has focused on the analysis of plasma lipids such as cholesterol, triacylglyceride, and phospholipids [8], [9]. Reports seldom indicate specific fatty acids and total cholesterol in erythrocytes as a predictor of cardiovascular events [10], [11], [12]. Additionally, erythrocytes are involved in reverse cholesterol transport (RCT), particularly in the low high-density lipoprotein (HDL) state [13]. Given the relatively long life (approximately 120 days) of erythrocytes, any change in the lipid profile of erythrocyte membrane may reflect pathophysiologic changes associated with disease progression.

Few studies have reported on the comprehensive assessment of the metabolome and lipidome of RBCs [14], [15], [16], especially in the scenario of HF. The aim of this study was to identify lipid profiles of HF erythrocytes using high-throughput liquid chromatography time-of-flight mass spectrometry (LC-TOF-MS). Our findings suggested that the erythrocyte lipid profiles of patients with HF were significantly different from those of normal subjects. The levels of phosphatidylcholines (PCs), phosphatidylethanolamines (PEs), and sphingomyelins (SMs) decreased in HF erythrocytes. However, the levels of lysoPCs, lysoPEs, and ceramides increased in these cells. Of these lipids, 7-ketocholesterol (7KCh) accumulated in the erythrocytes of patients with HF. This accumulation may be of significance as a potential discriminator and as a player in the pathogenesis of HF. At molecular level, we demonstrated that intracellular 7KCh accumulation caused reactive oxygen species (ROS) formation and cardiomyocyte death, which was probably mediated through ATP4/CHOP pathway.

## 2 Materials and methods

Chemicals were purchased from Sigma-Aldrich (St. Louis, MO), unless otherwise stated. Antibodies to ATF-4 (11815; Cell Signaling Technology, Danvers, MA, USA), CHOP (sc-7351; Santa Cruz Biotechnology, Inc., Dallas, TX, USA), LC3A/B (12741; Cell Signaling Technology, Danvers, MA, USA), Cleaved Caspase-3 (9664; Cell Signaling Technology, Danvers, MA, USA), Caspase-3 (9662; Cell Signaling Technology, Danvers, MA, USA), Actin (A5441, A2103; Sigma-Aldrich, St. Louis, MO, USA), and 7-ketocholesterol (Clone #35 A, Japan Institute for the Control of Aging, Shizuoka, Japan) were purchased from respective vendors.

### 2.1 Blood sample collection and erythrocyte preparation

Patients with HF were classified as stages A, B, and C according to the American College of Cardiology and the American Heart Association Heart Failure (ACC/AHA HF) classification system [17]: patients in stage A were at high risk and asymptomatic but did not have structural heart disease; patients in stage B had structural heart disease but were asymptomatic; and patients in stage C had been hospitalized due to acute or decompensated chronic HF. Patients aged 39–85 years were enrolled in this study. Patients with hypothyroidism, decompensated liver cirrhosis, systemic lupus erythematosus, or serum creatinine of >3 mg/dL were excluded. Informed consent was obtained from all patients. The study was designed and performed in accordance with the principles of the Declaration of Helsinki and with approval from the Ethics Review Board of Chang Gung Memorial Hospital.

Blood samples were collected in EDTA-containing tubes before the patients were discharged from the hospital. RBCs were prepared for LC-MS analysis. The plasma level of B-type natriuretic peptide (BNP) was measured in triplicate with the Triage BNP Test (Biosite, San Diego, CA). The precision, analytical sensitivity and stability characteristics of the assay were previously described [18]. Measurements of kidney function and other biochemical parameters were conducted in the Central Core Laboratory of Chang Gung Memorial Hospital at Keelung.

### 2.2 Lipidomics analysis by LC-MS

For human erythrocyte lipids, a modified method was employed [19]. In brief, 100 μL of erythrocyte lysates was transferred to a glass tube, and 100 μL of water was added. The sample was vortexed and then placed on ice for 15 min. After 1.1 mL of isopropanol was added, the sample was vortexed 4 times for 30 s. Approximately 700 μL of chloroform was added, and the sample was vortexed 4 times for 30 s again. It was subsequently centrifuged at 700×*g* for 30 min at 4 °C. The supernatant was transferred to a new tube, dried under nitrogen gas, and stored at −80 °C. Prior to analysis, the sample was dissolved in 500 μL of isopropanol/acetonitrile/water (2:1:1).

For lipid separation, an ACQUITY CSH C18 (2.1 mm × 100 mm, 1.7 µm) column was used. The column temperature was set at 55 °C and the flow rate at 400 μL/min. Mobile phase buffer A was acetonitrile/water (60/40) with 10 mM ammonium formate and 0.1% formic acid, whereas buffer B was isopropanol/acetonitrile (90:10) with 10 mM ammonium formate and 0.1% formic acid. The initial LC gradient conditions were 40% buffer B, increasing to 70% B within 12.1 min, and then to 99% B for 6 min before re-equilibration for 2 min at 40% B. The lyophilized sample was diluted with 500 μL of isopropanol/acetonitrile/water (2:1:1). Each sample was analyzed in triplicate. MS was performed on a Waters QTOFMS (SYNAPT G1 HDMS, Waters MS Technologies, Manchester, UK) operating in the positive or negative ion mode. The desolvation gas was set at 800 L/h at a temperature of 400 °C; the cone gas was set at 25 L/h; and the source temperature was set at 100 °C. The capillary voltage and cone voltage were set to 3000 and 35 V, respectively. Leucine encephalin was used as the lock mass standard (an [M+H]^+^ ion at 556.2771 Da in electrospray ionization (ESI) positive mode; an [M+H]^-^ ion at 554.2615 Da in ESI negative mode).

MS data were processed using MassLynx V4.1 and Progenesis QI software (Waters Corp., Milford, Massachusetts, USA). The intensity of each mass ion was normalized with respect to the total ion count to generate a data matrix that included the retention time, *m/z* value, and normalized peak area. The multivariate data matrix was analyzed using SIMCA-P software (version 13.0, Umetrics AB, Umea, Sweden).

Significant metabolites were subjected to a database search using an in-house database, the Human Metabolome Database (HMDB) (http://www.hmdb.ca/), or the METLIN Metabolomics Database (metlin.scripps.edu/index.php). For the identification of specific metabolites, MS/MS spectra were collected and confirmed by comparison with the spectra of chemical standards or by searching the HMDB and METLIN databases.

### 2.3 Quantification of erythrocyte and plasma 7KCh by LC-MS-MS

Erythrocyte 7KCh was extracted using a modified method [19] as described above. A sample was dissolved in isopropanol: acetonitrile:water (2:1:1 (v/v/v)) containing the deuterated forms of 7KCh-D7 and cholesterol-D6, which were employed as internal standards for quantification. For analysis of total-form sterols and free-form sterols in plasma, plasma samples (10 μL) were preincubated with or without cholesteryl ester hydrolase at 37 °C for 15 min in 10 mM phosphate buffer containing taurocholic acid [20]. The samples were extracted with 500 μL of methanol containing cholesterol-D6 and 7-KCh-D7, and then centrifuged at 12,000×*g* at 4 °C for 30 min. The resulting supernatant was collected in a new microtube and dried with nitrogen. The sample was dissolved in 100 μL of 100% methanol and was centrifuged at 12,000×*g* at 4 °C for 30 min for 7KCh, cholesterol, lanosterol, lathosterol, and 7-dehydrocholesterol quantification.

All the samples were analyzed using ultra-high-performance LC coupled with Xevo TQ-S MS (Waters Corp.) as previously described with modifications [21]. MS with atmospheric pressure chemical ionization (APCI) was performed in positive-ion multiple-reaction-monitoring mode. The optimized parameters were as follows: corona discharge current at 1 μA; probe temperature at 600 °C; source temperature at 150 °C; and gas flow at 800 L/h. Chromatographic separation was achieved on a pentafluorophenyl (PFP) column (100 mm×2.1 mm; with particle size of 1.8 µm; Waters Corp.) at 25 °C with eluent A (acetonitrile: water 25:75 (v/v) with 0.1% formic acid) and eluent B (methanol); the flow rate was set at 0.3 mL/min. The gradient profile was as follows: isogradient 70% B, 1 min; linear gradient 70–75.7% B, 8 min; linear gradient 75.7–100% B, 1 min; and 100% B, 2 min. The column was then re-equilibrated for 8 min for the next analysis.

### 2.4 Cell culture and viability assay

HL-1 atrial myocytes were cultured in fibronectin–gelatin-coated flasks containing Claycomb medium, supplemented with 10% fetal bovine serum, 100 U/mL of penicillin, 100 μg/mL of streptomycin, 2 mM L-glutamine, and 0.1 mM norepinephrine in a humidified atmosphere containing 5% CO_2_ at 37 °C, as previously described [22]. For 7KCh treatment, 2 × 10^4^ cells were incubated with 10, 20, and 50 µM 7KCh for the indicated periods. To mimic the delivery of 7KCh from erythrocyte to cardiomyocytes, we prepared erythrocyte ghost and loaded it with 7KCh. The packed erythrocytes were hemolyzed in hypotonic phosphate buffer (2.5 mM NaH_2_PO_4_, 2.5 mM Na_2_HPO_4_, 1 mM EDTA, pH 8) and centrifuged at 16,000 g to precipitate the ghosts. The erythrocyte membrane was incubated with 7KCh or cholesterol at 37 °C for 4 h, after which the membrane-bound 7KCh or cholesterol in the ghost pellet was diluted to the indicated concentrations for a viability test. Cell viability was determined by a neutral red assay, as previously described [23].

### 2.5 Cytometric analysis of ROS

ROS formation was analyzed quantitatively by cytometric analysis. In brief, HL-1 cells were loaded with 5 μM MitoSOX red or 5 μM H_2_DCFDA for 30 min at 37 °C, washed twice with PBS, and trypsinized for flow cytometric analysis as previously described [24]. The mean fluorescence intensity (MFI) of the fluorescence of oxidized MitoSOX red or of DCF fluorescence was quantified using CellQuest Pro software (Becton Dickinson, CA, USA).

### 2.6 Immunostaining for7KCh

HL-1 cells were fixed and permeabilized with 4% paraformaldehyde, 0.1% Triton-X100 in PBS for 2 h. After PBS wash, cells were treated with PBS containing 0.1% Triton X-100 and 5% BSA for 1 h at room temperature. Cells were stained with primary antibody of 7KCh (1:100; Clone #35 A, Japan Institute for the Control of Aging, Shizuoka, Japan) overnight at 4 °C. Secondary antibody, conjugated to Alexa-488 (Thermo Fisher Scientific Inc., Waltham, MA, USA), was added at a 1:200 dilution and incubated for 1–2 h. Hoechst 33342 (Thermo Fisher Scientific Inc., Waltham, MA, USA was used to stain cellular nuclei.

### 2.7 Western blotting assay

SDS-PAGE and western blotting were performed as previously described [25]. The cells were rinsed with cold PBS, scraped and collected by centrifugation. Cells were immediately lysed in lysis buffer (20 mM Tris. HCl (pH 8), 1% Triton X-100, 137 mM NaCl, 1.5 mM MgCl_2_, 10% glycerol, 1 mM EGTA, 50 mM NaF, 1 mM Na_3_VO_4_, 10 mM β-glycerophosphate, 1 mM PMSF, 1 μg/mL leupeptin, 1 μg/mL aprotinin). Protein concentration of the lysate was determined by the Bradford method. The sample was analyzed by SDS-PAGE and immunoblotting with antibodies to transcription factor ATF4, CHOP, LC3A/B, cleaved caspase 3, caspase 3 and actin according to manufacturers’ instructions.

### 2.8 Statistical analyses

To maximize the number of differentially abundant metabolites among groups, the orthogonal projection to latent structures discriminant analysis (OPLS-DA) model was applied using SIMCA-P software (version 13.0, Umetrics AB, Umea, Sweden). The variable importance in the projection (VIP) value of each variable in the model was calculated to indicate its contribution. S-plots were constructed from OPLS-DA data. Metabolites were chosen based on their contribution to the variation and correlation within the data set of the control and the stage C HF groups. The VIP values of variables greater than 1.0 are considered significantly different.

Results are expressed as the mean ± SD for continuous variables and as the number (percentage) for categorical variables. Data were compared by two-sample *t*-tests. A p value of < 0.05 was considered significant.

## 3 Results

### 3.1 Baseline characteristics

Initially, a total of 115 subjects were enrolled in this study, comprising 28 normal controls and 87 patients at stages A (n = 29), B (n = 29), and C (n = 29). The baseline characteristics and laboratory data are shown in Table 1. Notable differences in these parameters were observed between patients at stages A, B, and C. Compared with the normal controls, patients at stage C had remarkably higher glucose, creatinine, and BNP levels, but they had lower total cholesterol, low-density lipoprotein (LDL) cholesterol, HDL cholesterol, sodium, hemoglobin and albumin levels, and lower estimated glomerular filtration rate. The percentage of male subjects was higher in the patient groups. For the untargeted lipid profile study, sex- and age-matched samples from 10 controls and 29 patients with HF at stage A (n = 10), stage B (n = 9), and stage C (n = 10) were chosen (Supplementary Table S1).

**Table 1:** Baseline characteristics of the study populations.

**Variable**	**Normal**	**Stage A**	**Stage B**	**Stage C**	**p-value**	**p-value**
	**(n=28)**	**(n=29)**	**(n=29)**	**(n=29)**	**(ANOVA)**	**(N vs. C)**
**Age (years)**	57.93±4.35	57.07±10.97	62.24±10.06	57.14±15.42	0.2299	0.7924
**Male (%)**	32 (9)	62 (18)	83 (24)	72 (21)	2.07E−05	0.0002
**LVEF (%)**	73.36±6.75	73.09±6.09	59.11±13.05	32.31±12.82	1.02E−30	3.19E−18
**Biochemistry parameters**						
**BNP (pg/mL)**	8.26±4.91	51.15±57.08	115.76±126.38	729.52±592.54	4.07E−12	4.17E−07
**Glucose (mg/dL)**	98.86±12.19	121.17±63.74	128.67±51.47	115.28±26.73	0.0706	0.0047
**HbA1c (%)**	5.56±0.25	6.03±0.93	6.56±1.18	5.96±0.46	0.0002	0.0002
**BUN (mg/dL)**	12.21±2.92	14.83±5.16	17.41±8.21	20.83±8.69	3.22E−05	1.43E−05
**Creatinine (mg/dL)**	0.70±0.17	0.99±0.29	1.00±0.24	1.08±0.30	8.12E−07	4.12E−07
**eGFR (mL/min/1.73** **m^2^)**	98.00±15.86	76.75±22.24	76.21±21.19	70.90±19.54	5.15E−06	4.25E−07
**Sodium (mEq/L)**	140.61±1.23	140.18±2.41	139.45±2.00	227.20±325.96	0.1149	0.1636
**Cholesterol (mg/dL)**	223.11±39.28	186.68±34.92	176.78±35.89	167.89±47.66	5.43E−06	1.65E−05
**Triglyceride (mg/dL)**	95.32±54.56	152.56±106.25	148.41±77.49	109.11±63.21	0.0143	0.3862
**LDL-cholesterol (mg/dL)**	146.96±34.23	120.76±65.28	105.52±36.11	102.76±38.13	0.0011	2.93E−05
**HDL-cholesterol (mg/dL)**	57.00±12.60	47.41±15.11	41.39±12.29	43.21±17.26	0.0006	0.0011
**Albumin (g/dL)**	4.46±0.21	4.13±0.47	3.97±0.43	3.98±0.49	5.26E−05	0.0001
**CBC parameters**						
**WBC (10^3^/mm^3^)**	6.24±1.46	7.13±2.48	8.78±3.85	8.44±2.56	0.0019	0.0002
**RBC (10^6^/mm^3^)**	4.70±0.45	4.49±0.62	4.59±0.69	4.55±0.78	0.6550	0.3656
**Hemoglobin (g/dL)**	13.83±1.35	13.27±1.80	14.05±2.04	13.73±2.15	0.4402	0.8374
**HCT (%)**	41.20±3.29	39.27±4.61	41.13±5.45	41.25±5.72	0.3396	0.9644
**Albumin (g/dL)**	4.46±0.21	4.13±0.47	3.97±0.43	3.98±0.49	5.26E−05	0.0001

LVEF, left ventricular ejection fraction; BNP, B-type natriuretic peptide; HbA1c, Hemoglobin A1c; BUN, Blood urea nitrogen; eGFR, estimate glomerular filtration rate; LDL-cholesterol, low density lipoprotein-cholesterol; HDL, high density lipoprotein-cholesterol; WBC, white blood cells; RBC, red blood cells; HCT, hematocrit.

### 3.2 Changes in lipidomes of erythrocytes in patients with heart failure

**Fig. 1:**
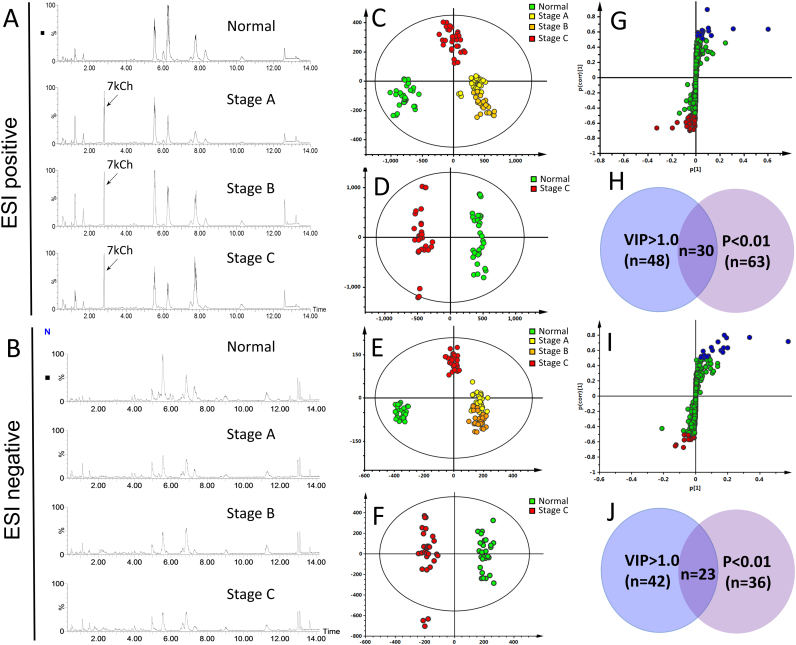
*Liquid chromatography time-of-flight mass spectrometry-based lipidomics analysis of heart failure (HF) erythrocytes.* Erythrocytes from normal control subjects and patients with stages A, B, and C were isolated for time-of-flight mass spectrometry analysis in electrospray ionization (ESI) positive and negative modes. Basal peak chromatograms of patients with HF at different stages and normal control subjects were obtained in ESI positive (A) and negative (B) modes, respectively. The molecular features were identified in samples (n = 39) by using Progenesis QI software and further data processing and statistical analysis were performed through SIMCA-P. Orthogonal partial least squares discriminant analysis (OPLS-DA) was performed for all samples, and the score plots for data sets obtained in ESI positive (C) and negative modes (E) are shown. The data for normal control and patients with stage C were reanalyzed using OPLS-DA, and the score plots for data sets obtained in ESI positive (D) and negative modes (F) are shown. Metabolites with significant differences in abundance in ESI positive and negative modes between normal control and patients with stage C are presented in S-plots (G and I), respectively. The Venn diagrams of the features obtained in ESI positive (VIP > 1.0 and p < 0.01) and ESI negative (VIP > 1.0 and p < 0.01) modes are shown in panels H and J, respectively. The normal control (n = 10), stage A (n = 10), stage B (n = 9), and stage C (n = 10) groups are marked in green, yellow, orange, and red, respectively. The ellipse shown in the model represents the Hotelling T2 with 95% confidence.

To investigate whether the erythrocyte lipid composition differs between patients with HF and normal controls, 39 RBC samples were subjected to untargeted metabolomic analysis using LC-TOF-MS. The typical spectra of erythrocyte extracts were obtained in positive (Fig. 1A) and negative (Fig. 1B) ion modes. After data processing with Progenesis QI, 457 and 438 molecular features obtained in ESI positive and negative modes were extracted, respectively. The data were subjected to SIMCA-P analysis. The derived OPLS-DA score plot showed remarkable separation between the control, stage A, stage B, and stage C groups for data sets obtained in both ESI positive and negative modes (Fig. 1C, E). To identify lipophilic metabolites discriminating between the samples from the normal controls and those from patients in group C, we reanalyzed these data sets. The OPLS-DA plots (Fig. 1D, F) and S-plots (Fig. 1G, I) for the data sets obtained in both ESI positive and negative modes are shown herein. From the S-plots, features with a p (corr) value of >0.5 or <−0.5 were selected. We observed that 30 and 23 metabolites acquired in both ESI positive and negative modes had VIP scores of >1.0 and revealed significant differences (p < 0.01) between the patients in stage C and the healthy controls (Fig. 1H, J). Clearly, sterols, phospholipids, and ceramides are important discriminators of the normal controls and patients in stage C (Supplementary Tables S2 and S3). The levels of lysophospholipids (such as lysoPC and lysoPE), ceramides, and oxysterols (such as 7KCh) were higher in the HF erythrocytes than in the control erythrocytes. By contrast, the levels of phospholipids (such as PCs, PEs, and sphingomyelins) decreased in the HF erythrocytes, compared with those of the normal cells (Table 2).

**Table 2:** The potential biomarkers identified for discriminating patients with HF from normal controls.

**No.**	**Metabolites**	**Fold change**			**p (corr)**	**p-value ANOVA**	**p-value (N vs. C)**	**VIP (N vs. C)**	**Trend of HF**
		**Log2 (A/N)**	**Log2 (B/N)**	**Log2 (C/N)**					
**1**	LysoPC C16:0	0.86	0.97	0.67	0.64	1.19E-08	1.67E-07	2.52	Increased
**2**	LysoPC C18:1	1.23	1.44	1.44	0.70	2.62E-10	6.26E-08	1.02	Increased
**3**	LysoPE C16:0	3.43	3.77	3.18	0.51	9.75E-09	2.39E-05	1.03	Increased
**4**	LysoPE C18:0	1.00	1.20	0.86	0.67	7.06E-10	2.70E-08	1.59	Increased
**5**	7-Dehydrocholesterol	3.28	3.57	2.76	0.65	9.22E-15	3.71E-07	1.62	Increased
**6**	7-Ketocholesterol	5.23	5.58	4.60	0.67	7.48E-18	3.59E-07	6.87	Increased
**7**	Cer C22:0	0.81	0.74	1.01	0.57	4.69E-06	1.66E-06	1.57	Increased
**8**	Cer C24:0	1.06	0.97	1.46	0.65	2.50E-09	2.49E-08	2.69	Increased
**9**	Cer C24:1	0.50	-0.49	1.61	0.53	1.27E-09	5.44E-05	1.33	Increased
**10**	LysoPE C20:4	-2.31	-1.82	-1.21	-0.53	2.95E-11	4.75E-05	1.64	Decreased
**11**	PC C34:0	-0.94	-0.86	-0.97	-0.61	7.37E-12	3.47E-07	1.32	Decreased
**12**	PC C34:2	-1.57	-1.10	-1.34	-0.63	8.53E-17	7.58E-08	12.87	Decreased
**13**	PC C36:1	-1.43	-1.72	-1.03	-0.55	1.74E-11	1.61E-06	2.89	Decreased
**14**	PC C36:2	-1.09	-0.98	-1.21	-0.64	2.07E-18	1.76E-07	6.72	Decreased
**15**	PC C36:3	-2.25	-2.62	-0.96	-0.61	1.59E-24	2.03E-07	2.29	Decreased
**16**	PC C36:4	<-6.00	<-6.00	-2.85	-0.62	1.69E-16	7.48E-07	1.14	Decreased
**17**	PE C34:1	-3.54	-5.06	-3.23	-0.51	5.45E-09	1.66E-05	1.24	Decreased
**18**	PE C34:2	-2.76	-2.88	-2.20	-0.77	2.07E-21	2.29E-09	3.98	Decreased
**19**	PE C36:1	-1.72	-1.46	-1.44	-0.52	1.72E-07	0.00027	1.42	Decreased
**20**	PE C36:2	-2.13	-2.92	-1.30	-0.60	3.49E-14	2.87E-05	2.01	Decreased
**21**	PE C36:3	-3.82	-4.19	-2.64	-0.80	4.90E-30	1.22E-10	3.78	Decreased
**22**	PE C36:4	-2.80	-3.32	-1.33	-0.60	1.46E-19	4.69E-07	3.37	Decreased
**23**	SM C24:2	-0.75	-0.69	-0.59	-0.65	7.55E-18	1.75E-08	2.25	Decreased
**24**	SM C24:1	-1.21	-0.98	-1.56	-0.64	3.37E-08	1.84E-07	2.00	Decreased

PC, phosphatidylcholine; PE, phosphatidylethanolamine; Cer, ceramide; SM, sphingomyelin; VIP, The variable importance in the projection.

### 3.3 Erythrocyte and plasma 7KCh levels in patient with heart failure

**Fig. 2:**
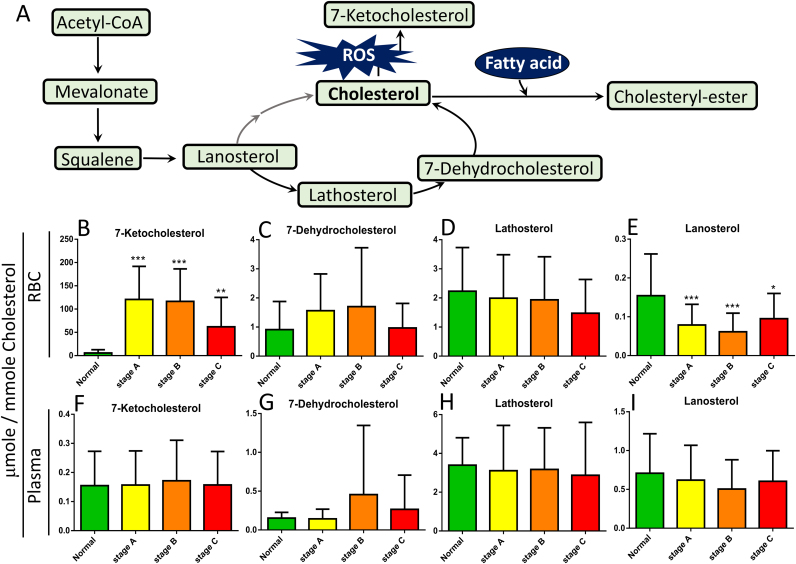
*Quantification of the free forms of 7KCh and other sterols in plasma and erythrocytes from patients with HF and controls.* Erythrocyte samples were extracted for free-form sterols, and the levels of cholesterol, lanosterol, lathosterol, 7-dehydrocholesterol, and 7KCh (7-ketocholesterol) were quantified using a LC-MS-MS. The levels of 7KCh, lanosterol, lathosterol, and 7-dehydrocholesterol were normalized to the level of cholesterol (per mmole). All samples from the normal control subjects (NC, n = 28) and patients with HF in stages A (n = 29), B (n = 29), and C (n = 29) are represented in panels B, C, D, and E, respectively. The free-form sterols in the corresponding plasma samples were determined, and the levels were also normalized to the level of cholesterol (per mmole) and are shown in panels F, G, H, and I. A schematic showing the cholesterol biosynthesis pathway (A). Data are expressed as 7KCh/Cholesterol (μmole/mmole). Data are means ± SD; *p < 0.05, **p < 0.01, ***p < 0.001 for patients with stages A, B, or C vs. controls.

**Fig. 3:**
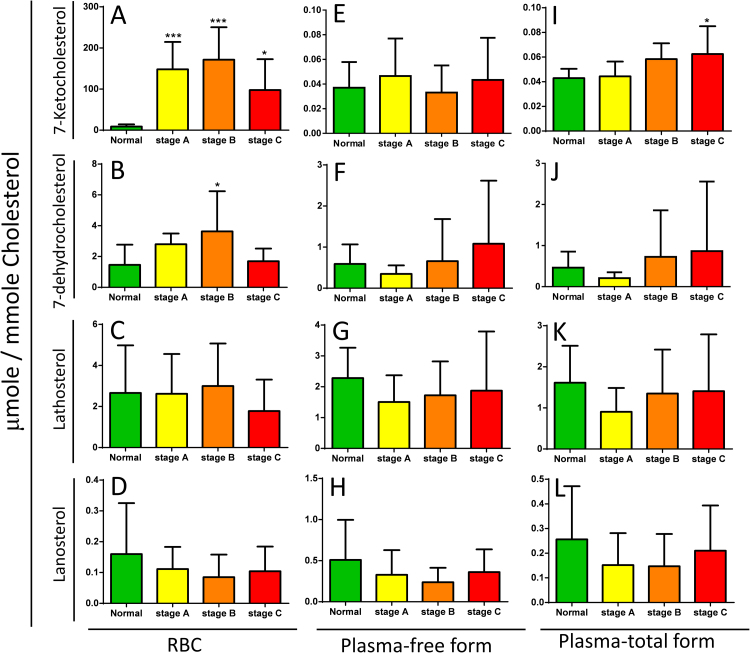
*Quantification of the total forms of 7KCh and other sterols in plasma from HF patients.* For total cholesterol (free cholesterol plus cholesteryl ester) quantification, plasma samples were pre-treated with cholesteryl ester hydroxylase as described in *Materials and methods* section. The sterols were extracted, and the levels of cholesterol, lanosterol, lathosterol, 7-dehydrocholesterol and 7KCh (7-ketocholesterol) in sample were quantified by LC-TQ mass spectrometer. The levels of 7KCh, lanosterol, lathosterol, 7-dehydrocholesterol in samples from normal control subjects (NC, n=10) and patients with HF in stage A (n=10), stage B (n=9), and stage C (n=10) were represented in panel I, J, K, and L, respectively. The free form of sterols in the corresponding plasma samples were determined, and the levels were shown in panel E, F, G, and H. For comparison, the free form of sterols in the corresponding erythrocyte samples were shown in panel A, B, C, and D. Data are expressed as 7KCh/Cholesterol (μmole/mmole). Data are means ± SD; *p<0.05, ***p<0.001, group of stage A, B, or C patients vs. control group.

The distribution of 7KCh between plasma and erythrocytes was reported for healthy volunteers [26]. Of the samples from the normal controls and patients in the current study, the 7KCh level was substantially lower than the levels of lanosterol, lathosterol, and 7-dehydrocholesterol (intermediates in cholesterol biosynthetic pathway) in plasma. By contrast, the 7KCh level was significantly higher than the levels of these metabolites in erythrocytes (Fig. 2, Fig. 3).

We observed significant increases in the levels of erythrocyte 7KCh in patients at stages A–C, suggesting that erythrocyte 7KCh can be used as a biomarker for the early identification of subjects at risk of HF. To test such a possibility, we determined the levels of cholesterol, lanosterol, lathosterol, 7-dehydrocholesterol, and 7KCh in erythrocytes and plasma from patients with HF and normal control subjects. The levels of lanosterol, lathosterol, 7-dehydrocholesterol, and 7KCh were normalized to that of cholesterol. The level of 7KCh in the erythrocytes was higher in patients in stages A, B, and C than in the controls. The levels of 7KCh in the control subjects and in patients in stages A, B, and C were 5.75 ± 6.88, 120.79 ± 70.93, 116.64 ± 69.69, and 62.03 ± 63.11 µmole per mmole of cholesterol, respectively (Fig. 2B). Cholesterol exists as free and ester forms in plasma, and the ester form is the major storage and transport forms carried by lipoproteins. We observed no difference in the plasma level of 7KCh between the normal controls and various patient cohorts (Fig. 2F). When total cholesterol (free cholesterol plus cholesteryl ester) was considered, the plasma level of 7KCh was slightly higher in stage C patients than in the normal controls (Fig. 3I).

### 3.4 Intracellular 7KCh accumulation enhances ROS production and reduces the viability of cardiomyocytes

**Fig. 4:**
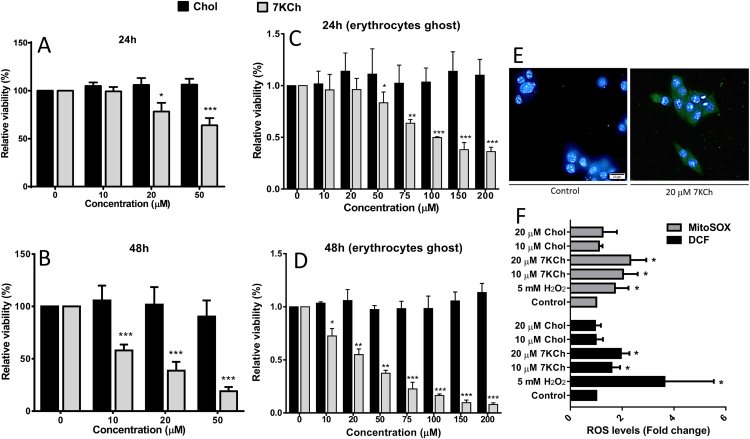
*Viability reduction and ROS generation in 7KCh accrued HL-1 cells.* HL-1 cells (5 × 10^4^/well) were treated with the indicated concentrations (0, 10, 20, 50 μM) of 7KCh and cholesterol (Chol) for 24 h (A) and 48 h (B), respectively. In addition, cells were treated with different concentrations (0, 10, 20, 50, 75, 100, 150, 200 μM) of 7KCh- or cholesterol-loaded erythrocyte ghost for 24 h (C) and 48 h (D), respectively. The viabilities of the treated cells were determined, and are expressed as percentage of that of untreated HL-1 cells. Data are means ± SD, n = 3; *p < 0.05, **p < 0.01, ***p < 0.001 for 7KCh-treated cells vs. untreated cells. (E) Immunostaining for 7KCh in HL-1 cells. HL-1 cells were untreated (control) or treated 20 μM 7KCh, and stained with primary antibody to 7KCh and secondary antibody conjugated to Alexa-488 (green). Hoechst 33342 (blue) was used to stain cellular nuclei. A representative experiment out of three is shown. (F) 7KCh induces ROS generation. HL-1 cells were treated with 10, 20 μM 7KCh or cholesterol (Chol) for 24 h, stained with H_2_DCFDA (green) and MitoSOX red (red), and analyzed using flow cytometry. H_2_O_2_-treated cells were positive control. The MFI was determined, and is expressed as fold change relative to that of untreated HL-1 cells. Data are means ± SD, n = 3; *p < 0.05 for 7KCh-treated cells vs. untreated cells.

To examine whether 7KCh impairs the physiology of cardiomyocytes, we tested the viability of HL-1 cells receiving 7KCh treatment. As the level of 7KCh in healthy volunteers was 1–2 μM [26] and the blood levels of 7KCh in patients with HF were 10- to 30-fold higher than those of the normal controls (Fig. 2), 7KCh was used at concentrations ranging from 10 μM to 50 μM so as to mimic the pathophysiological condition. 7KCh caused a dose-dependent reduction in the viability of HL-1 cells. As a control, cholesterol treatment did not affect the viability of the cells (Fig. 4A, B). A plausible possibility is that 7KCh in erythrocytes can be released to affect the viability of cardiomyocytes. To test this possibility, we loaded the erythrocyte membrane with 7KCh and incubated cardiomyocytes with the 7KCh-laden membrane. Upon treatment, the viability of the HL-1 cells declined in a dose-dependent manner (Fig. 4C, D). These results suggest that 7KCh can be delivered from erythrocytes to cause cellular damage.

To ensure whether 7KCh accumulates in HL-1 cells, we performed an immunofluorescence assay with anti-7KCh antibody to detect the intracellular level of 7KCh in HL-1 cell (Fig. 4E). The 7KCh accumulated in cytosol of HL-1 cell upon 24 h treatment with 20 μM 7KCh.

To determine whether 7KCh causes ROS generation in HL-1 cells, we treated cells with 7KCh for 24 h, stained with H_2_DCFDA, and analyzed them using flow cytometer. As expected, the DCF fluorescence increased in cells treated with 10 μM and 20 μM 7KCh (Fig. 4F). H_2_O_2_-treated HL-1 cells served as positive control. It was consistent with the results for cells stained with MitoSOX Red, a mitochondrion-specific probe for ROS. The 7KCh-treated HL-1 cells showed higher ROS production than positive control (i.e. HL-1 cells treated with 5 mM H_2_O_2_) (Fig. 4F).

### 3.5 7KCh induces cell death through activation of transcription factor 4 (ATF4) pathway

**Fig. 5:**
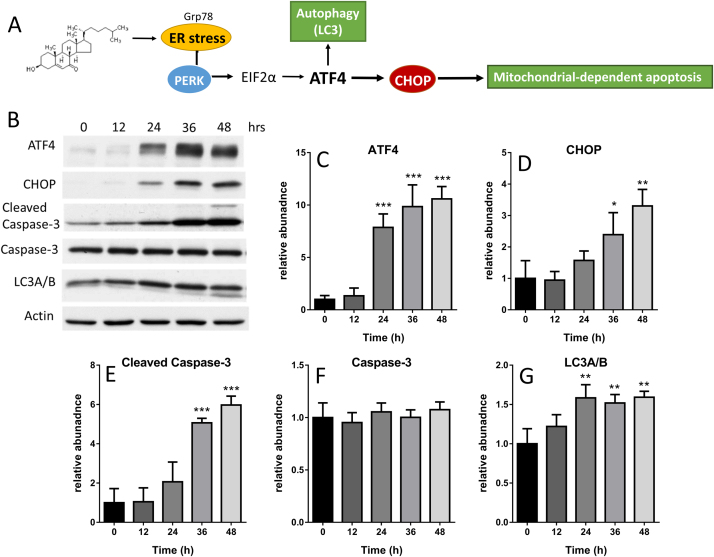
*7KCh activates transcription factor 4 (ATF4) pathway.* (A) A schematic diagram showing the proposed effect of 7KCh on ATF4/CHOP pathway. (B) HL-1 cells were treated with 10 μM 7KCh for the indicated periods (0, 12, 24, 36, 48 h), and the cell lysate was analyzed for the levels of ATF4, CHOP, and caspase 3, LC3 and actin (loading control) by immunoblotting with respective antibodies. A representative experiment out of three is shown. The relative intensities of ATF4 (C), CHOP (D), cleaved Caspase-3 (E), caspase-3 (F), and LC3A/B (G) were normalized to that of actin, and are expressed as fold change relative to those of untreated HL-1 cells. Data are mean ± SD, n=3. *p<0.05, **p<0.01, ***p<0.001 vs. untreated cells.

ATF4 is induced by various stressors, such as endoplasmic reticulum (ER), amino acid deprivation, and oxidative stress [27]. ER stress and oxidative stress accentuate each other, which have been shown to activate apoptotic signaling in vitro and in vivo models [28]. To test the possibility that 7KCh acts through activation of ATF4, we examined the expression level of ATF4 in 7KCh-treated HL-1 cells. The ATF4 level increased in a time-dependent manner (Fig. 5B, C). Moreover, the levels of CHOP and active caspase 3 increased significantly at 36 h after treatment (Fig. 5B, D, E), whereas that of LC3A/B increased slightly during the same period (Fig. 5B, G). These findings suggest that induction of ATF4 and CHOP contribute to death of cardiomyocytes.

## 4 Discussion

The lipidomes of erythrocytes from patients with HF were significantly different from those of normal controls. Moreover, 7KCh, lysoPCs, lysoPEs, and ceramides significantly increased in abundance in HF erythrocytes, whereas the levels of PCs, PEs, and SMs significantly decreased. Of these lipids, 7KCh was best at discriminating between patients and controls, and may thus serve as a biomarker for early identification of individuals at risk of HF. Additionally, 7KCh may be implicated in death of cardiomyocytes and HF pathogenesis.

Chronic inflammation is associated with HF progression. A number of proinflammatory cytokines, such as tumor necrosis factor α, interleukin (IL)-1, and IL-6, were implicated in this process [29]. In general, chronic inflammation leads to increased oxidative stress and damage and probably accounts for some of the observed changes in HF erythrocytes. Oxidative stress induces phospholipase activity, which leads to a decline in phospholipid levels and an increase in lysophospholipids levels. Moreover, 7KCh, an oxidation product of cholesterol, accumulates as a consequence of oxidative stress. Previous studies have revealed that oxidative damage products, such as oxidized LDL and oxysterols, are found in patients with cardiovascular disease [30], [31]. 7KCh is considered an important metabolite for monitoring cardiovascular disease outcomes and mortality [32] as well as for predicting the incidence of cardiovascular disease events in general population [33]. Accumulation of 7KCh in HF erythrocytes suggests that 7KCh is a risk factor for HF, with a potential for clinical applications.

7KCh is mainly derived from oxidation of biomembranes and lipoproteins. Dietary intake of food oxysterols are not probably an important source of 7KCh, as dietary 7KCh can be rapidly metabolized to bile acid in liver and excreted [34], [35], [36]. Erythrocyte 7KCh may be derived from oxidation of membrane of erythrocytes. Moreover, elevation in erythrocyte 7KCh levels may be related to the changes in its catabolism in patients with HF. CYP7A1, the rate-limiting enzyme in bile acid biosynthesis, is the only hepatic enzyme known to be involved in 7KCh catabolism [37]. CYP7A1 deficiency causes premature atherosclerosis in humans [38], [39]. The extrahepatic metabolism of 7KCh occurs by esterification to fatty acids through cytosolic sterol O-acyltransferase (SOAT) and subsequent selective efflux to HDL [37]. HDL transfers esterified 7KCh back to liver for further catabolism. Relatively low SOAT1 and SOAT2 expression in heart tissue leads to poor esterification and accumulation of free-form 7KCh. A decrease in reverse cholesterol transport (RCT) via HDL ensues [37]. The situation is aggravated by lower plasma HDL content in HF patients. It has been recently shown that erythrocytes play an important role in RCT. Animal study has shown that erythrocytes acquired tritiated cholesterol from subcutaneously injected foam [13]. Also, erythrocytes can exchange cholesterol with lipoproteins [40]. It is likely that erythrocytes may take up 7KCh from peripheral tissues and/or from other lipoproteins. Erythrocytes can then transport 7KCh directly to liver for catabolism, or transfer it to HDL via the exchange mechanism. The latter process may be less effective in light of the decrease in plasma HDL in HF patients. An increase in 7KCh abundance in erythrocytes may reflect not only increased oxidative stress but also an ineffectual removal through RCT to HDL in HF patients.

7KCh causes cellular damage and induces oxidative stress in several types of cells, such as endothelial and cardiac cells [41], [42]. Exposure to 7KCh promotes inflammation [43], ER stress [44], [45], and lysosomal dysfunction [46]. 7KCh triggers autophagy through inhibition of Atg4B activity [44], and induces lysosomal dysfunction in vascular smooth muscle cells through an increase in oxidative stress [42]. Our findings show that 7KCh induces ROS formation and causes cardiomyocyte death, which is probably mediated by the ATF4/CHOP pathway. Previous studies have shown that ER stress leads to activation of PERK, phosphorylation of eukaryotic translation initiation factor 2, and subsequent translation of mRNAs, including ATF4. ATF4 itself can activate the transcription of CHOP, which is essential to ER stress-induced cell death [47], [48], [49], [50], [51], [52], [53].

**Fig. 6:**
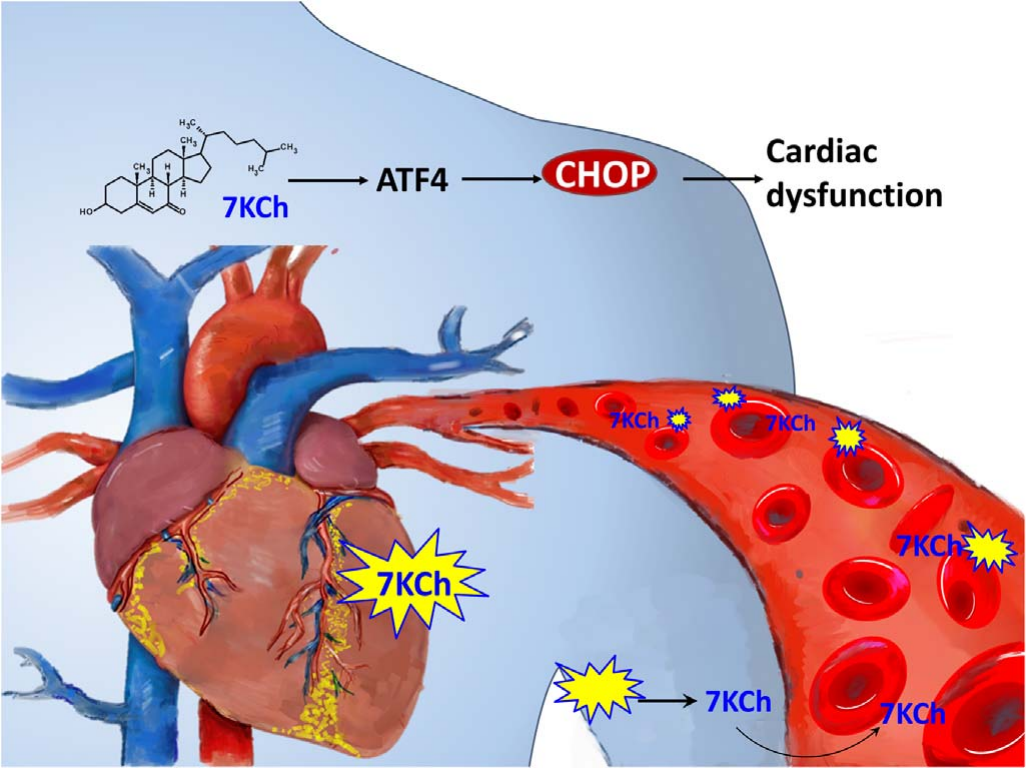
*A proposed model of the effect of erythrocyte 7KCh on cardiomyocytes.* Erythrocytes may transport 7KCh to cardiac tissue, and inflicts damage to cardiac cells.

Our findings may have interesting implications about the transport functions of erythrocytes. Erythrocytes may act as a transporter of substances other than oxygen. The free-form cholesterol of the erythrocyte plasma membrane can be bidirectionally exchanged with that of plasma lipoprotein and cellular plasma membrane. Erythrocytes were postulated to play a role in RCT, particularly in the low HDL state [13]. Our unpublished findings have shown an inverse correlation between erythrocyte 7KCh and plasma HDL in HF patients. Erythrocytes, in addition to HDL, may perform RCT function. Another intriguing possibility is that erythrocytes may transport 7KCh to cardiac tissue and inflict damage to cardiac cells. The proposed scheme is summarized in Fig. 6.

The current study has several limitations. One of the limitations pertains to the small cohort size of patients at different stages of HF. Although we demonstrated that 7KCh induces cardiomyocyte death, the evidence supporting the causal relationship between an increase in erythrocyte 7KCh and HF development is far from complete. An animal model is needed in further study to validate our findings.

In conclusion, blood 7KCh is concentrated in erythrocytes in patients with HF. 7KCh-laden erythrocyte ghost induces cardiomyocyte death, suggesting its involvement in the pathogenesis of HF. Moreover, our findings support the importance of erythrocyte 7KCh as a risk factor for HF.
