# Supplementary material for: Lipidomics reveals accumulation of the oxidized cholesterol in erythrocytes of heart failure patients
Source: Redox Biol. 2017 Oct 26;14:499–508. doi: 10.1016/j.redox.2017.10.020 (PMC5675899; doi:10.1016/j.redox.2017.10.020)
Supplement: Supplementary file 1 — Supplementary material [file mmc1.docx]

**Supplemental Material**

**Lipidomics reveals accumulation of the oxidized cholesterol in erythrocytes of heart failure patients**

Hsiang-Yu Tang^1^, Chao-Hung Wang^2,3^, Hung-Yao Ho^4,5^, Pei-Ting Wu^6^, Chun-Ling Hung^6^, Cheng-Yu Huang^1^, Pei-Ru Wu^7^, Yung-Hsin Yeh^8^, Mei-Ling Cheng^1,5,7,*^

^1^Metabolomics Core Laboratory, Healthy Aging Research Center, Chang Gung University, Tao-yuan, Taiwan; ^2^Heart Failure Research Center, Division of Cardiology, Department of Internal Medicine, Chang Gung Memorial Hospital, Keelung, Taiwan; ^3^College of Medicine, Chang Gung University, Taoyuan, Taiwan; ^4^Department of Medical Biotechnology and Laboratory Science, College of Medicine, Chang Gung University, Taoyuan, Taiwan; ^5^Clinical Phenome Center, Chang Gung Memorial Hospital, Taoyuan, Taiwan; ^6^Graduate Institute of Biomedical Sciences, College of Medicine, Chang Gung University, Taoyuan, Taiwan; ^7^Department of Biomedical Sciences, College of Medicine, Chang Gung University, Taoyuan, Taiwan; ^8^Cardiovascular Division, Chang-Gung Memorial Hospital, Chang-Gung University College of Medicine, Chang-Gung University, Taiwan

***Corresponding author:**

Mei-Ling Cheng, Ph.D.

Department of Biomedical Sciences, College of Medicine, Chang Gung University, No.259, Wenhua 1st Rd., Guishan Dist., Taoyuan City 33302, Taiwan (R.O.C.)

E-mail address: [chengm@mail.cgu.edu.tw](mailto:chengm@mail.cgu.edu.tw); Phone:886-3-2118244

**Supplemental Tables**

Supplementary Table S1. Baseline characteristics of the study populations

| Variable | Normal  (n=10) | Stage A  (n=10) | Stage B  (n=9) | Stage C  (n=10) | p-value  (ANOVA) | p-value  (N vs. C) |
| --- | --- | --- | --- | --- | --- | --- |
| Age (years) | 59.60±2.63 | 57.30±9.80 | 61.89±8.81 | 59.80±12.13 | 0.748 | 1.000 |
| Male (%) | 7 (70) | 8 (80) | 8 (89) | 7 (70) | 0.748 | 1.000 |
| LVEF (%) | 71.50±6.80 | 75.09±5.41 | 49.17±12.87 | 31.39±10.48 | 0.000 | 0.000 |
| Biochemistry parameters |  |  |  |  |  |  |
| BNP (pg/mL) | 9.04±7.49 | 41.96±39.40 | 163.80±182.77 | 712.31±515.79 | 0.000 | 0.000 |
| Glucose (mg/dL) | 106.60±13.94 | 103.20±16.28 | 142.89±54.57 | 125.90±38.81 | 0.060 | 0.598 |
| HbA1c (%) | 5.57±0.25 | 6.07±0.94 | 7.30±1.38 | 6.22±0.35 | 0.001 | 0.261 |
| BUN (mg/dL) | 13.00±3.30 | 14.90±5.02 | 23.22±10.66 | 21.20±9.24 | 0.014 | 0.091 |
| Creatinine (mg/dL) | 0.83±0.18 | 1.08±0.36 | 1.01±0.28 | 1.09±0.34 | 0.183 | 0.207 |
| eGFR (ml/min/1.73 m^2^) | 90.30±13.14 | 74.38±25.55 | 78.67±22.59 | 70.20±23.52 | 0.209 | 0.183 |
| Sodium (mEq/L) | 140.25±1.16 | 139.27±2.93 | 138.84±1.74 | 139.55±2.37 | 0.550 | 0.887 |
| Cholesterol (mg/dL) | 228.60±38.15 | 172.00±20.77 | 179.44±31.95 | 157.10±47.54 | 0.001 | 0.001 |
| Triglyceride (mg/dL) | 103.60±67.76 | 195.44±119.74 | 154.33±90.88 | 142.50±68.68 | 0.177 | 0.758 |
| LDL-cholesterol (mg/dL) | 153.10±28.77 | 92.37±19.92 | 107.67±30.38 | 91.18±38.80 | 0.000 | 0.000 |
| HDL-cholesterol (mg/dL) | 54.80±11.05 | 43.07±19.38 | 38.28±9.45 | 37.54±8.64 | 0.020 | 0.024 |
| Albumin (g/dL) | 4.46±0.29 | 4.04±0.42 | 4.00±0.39 | 4.05±0.57 | 0.083 | 0.168 |
| CBC parameters |  |  |  |  |  |  |
| WBC (10^3^/mm^3^) | 6.75±0.99 | 7.51±2.37 | 9.31±3.75 | 7.94±2.11 | 0.167 | 0.703 |
| RBC (10^6^/mm^3^) | 4.93±0.32 | 4.31±0.61 | 4.46±0.46 | 4.38±1.01 | 0.161 | 0.251 |
| Hemoglobin (g/dL) | 15.00±1.33 | 12.96±1.56 | 13.89±1.81 | 13.59±2.44 | 0.110 | 0.330 |
| HCT (%) | 43.86±3.43 | 38.12±4.18 | 40.40±4.40 | 40.42±7.07 | 0.099 | 0.422 |

LVEF, left ventricular ejection fraction; BNP, B-type natriuretic peptide; HbA1c, Hemoglobin A1c; BUN, Blood urea nitrogen; eGFR, estimate glomerular filtration rate; LDL-cholesterol, low density lipoprotein-cholesterol; HDL, high density lipoprotein-cholesterol; WBC, white blood cells; RBC, red blood cells; HCT, hematocrit.

Supplementary Table S2. Significantly changed metabolites in patients with heart failure were selected in ESI positive mode.

| No. | Metabolites | m/z | RT  (min) | Fold change | | | P (corr) | p-value  ANOVA | p-value  (N vs.C) | VIP  (N vs.C) |
| --- | --- | --- | --- | --- | --- | --- | --- | --- | --- | --- |
|  |  |  |  | **Log2**  **(A/N)** | **Log2**  **(B/N)** | **Log2**  **(C/N)** |  |  |  |  |
| Significantly increased metabolites in HF | | | | | | | | | | |
| 1 | LysoPC(18:1) (M+H) | 522.3585 | 1.26 | 1.23 | 1.44 | 1.44 | -0.7 | 2.62E-10 | 6.26E-08 | 1.023 |
| 2 | Cholesta-4,6-dien-3-one | 383.3332 | 4.53 | 2.02 | 2.22 | 1.64 | -0.69 | 2.78E-16 | 1.65E-08 | 1.120 |
| 3 | 7-Ketocholesterol (M+H) | 401.3506 | 2.79 | 5.23 | 5.58 | 4.60 | -0.67 | 7.48E-18 | 3.59E-07 | 6.869 |
| 4 | 7-Ketocholesterol (2M+Na) | 823.6670 | 2.79 | > 6 | > 6 | > 6 | -0.67 | 1.06E-17 | 2.56E-07 | 1.000 |
| 5 | LysoPC(16:0) (M+H) | 496.3517 | 1.20 | 0.89 | 0.89 | 0.67 | -0.66 | 1.89E-09 | 1.19E-07 | 4.005 |
| 6 | 7-Dehydrocholesterol (M+H-H_2_O) | 367.3384 | 2.77 | 3.28 | 3.57 | 2.76 | -0.65 | 9.22E-15 | 3.71E-07 | 1.615 |
| 7 | 2.56_790.5670 m/z | 790.5670 | 2.56 | > 6 | > 6 | > 6 | -0.62 | 1.65E-08 | 2.94E-06 | 1.097 |
| 8 | Hydroxy-cholesterol (M+Na) | 425.3417 | 3.05 | > 6 | > 6 | > 6 | -0.61 | 1.56E-13 | 3.10E-06 | 1.015 |
| 9 | 2.64_788.5513 m/z | 788.5513 | 2.64 | > 6 | > 6 | > 6 | -0.6 | 5.98E-17 | 5.19E-06 | 1.645 |
| 10 | 3.22_772.5568 m/z | 772.5568 | 3.22 | 2.70 | 3.05 | 2.23 | -0.6 | 5.05E-22 | 3.95E-06 | 1.240 |
| 11 | L-Carnitine (M+H) | 162.1125 | 0.53 | 1.46 | 1.33 | 1.55 | -0.59 | 9.19E-06 | 5.18E-06 | 3.361 |
| 12 | 2.01_825.6128 m/z | 825.6128 | 2.01 | > 6 | > 6 | > 6 | -0.59 | 2.05E-11 | infinity | 1.391 |
| 13 | 2.08_825.6129 m/z | 825.6129 | 2.08 | > 6 | > 6 | > 6 | -0.59 | 3.49E-11 | infinity | 1.151 |
| 14 | 2.75_788.5513 m/z | 788.5519 | 2.75 | > 6 | > 6 | > 6 | -0.59 | 5.87E-15 | 9.35E-06 | 1.708 |
| 15 | 2.78_622.4496 m/z | 622.4496 | 2.78 | > 6 | > 6 | > 6 | -0.57 | 6.67E-10 | infinity | 1.083 |
| 16 | 3.81_774.5719 m/z | 774.5719 | 3.81 | > 6 | > 6 | 5.93 | -0.54 | 1.33E-10 | 4.90E-05 | 1.367 |
| 17 | Ceramide 24:1 (M+H) | 670.6169 | 13.35 | 0.50 | -0.49 | 1.61 | -0.53 | 1.27E-09 | 5.44E-05 | 1.328 |
| 18 | 3.47_772.5565 m/z | 772.5565 | 3.47 | 2.94 | 3.30 | 2.35 | -0.51 | 4.28E-17 | 0.0002 | 1.009 |
| Significantly decreased metabolite in HF | | | | | | | | | | |
| 1 | L-Acetylcarnitine(M+H) | 204.1224 | 0.55 | -1.28 | -1.41 | -0.98 | 0.89 | 3.08E-43 | 2.19E-15 | 1.991 |
| 2 | SM 24:2 (M+H) | 811.6727 | 10.23 | -0.75 | -0.69 | -0.59 | 0.65 | 7.55E-18 | 1.75E-08 | 2.249 |
| 3 | PC(36:2) (M+H) | 786.6147 | 8.31 | -1.09 | -0.98 | -1.21 | 0.64 | 2.07E-18 | 1.76E-07 | 6.719 |
| 4 | PC(34:2) (M+H) | 758.5925 | 6.27 | -1.57 | -1.10 | -1.34 | 0.63 | 8.53E-17 | 7.58E-08 | 12.871 |
| 5 | PC(36:4) (M+H) | 782.577 | 5.25 | <-6 | <-6 | -2.85 | 0.62 | 1.69E-16 | 7.48E-07 | 1.143 |
| 6 | PC(36:3) (M+H) | 784.5939 | 6.45 | -2.25 | -2.62 | -0.96 | 0.61 | 1.59E-24 | 2.03E-07 | 2.292 |
| 7 | PC(34:0) (M+H) | 762.6082 | 9.95 | -0.94 | -0.86 | -0.97 | 0.61 | 7.37E-12 | 3.47E-07 | 1.32 |
| 8 | 3.17_790.5672 m/z | 790.5672 | 3.17 | -4.13 | -3.10 | -2.56 | 0.58 | 2.18E-17 | 7.85E-07 | 1.233 |
| 9 | 4.66_827.5844 m/z | 827.5844 | 4.66 | -1.21 | -1.40 | -0.71 | 0.57 | 4.24E-11 | 6.31E-06 | 1.029 |
| 10 | 4.80_827.5845 m/z | 827.5845 | 4.80 | -1.47 | -1.78 | -1.08 | 0.56 | 1.06E-09 | 1.13E-05 | 1.043 |
| 11 | PC(36:1) (M+H) | 788.6261 | 10.28 | -1.43 | -1.72 | -1.03 | 0.55 | 1.74E-11 | 1.61E-06 | 2.890 |
| 12 | PE(34:1) (M+H) | 718.5445 | 8.34 | -3.54 | -5.06 | -3.23 | 0.51 | 5.45E-09 | 1.66E-05 | 1.238 |

Metabolites of interest were extracted from S-plots constructed following OPLS-DA, and markers were chosen based on their p (corr) value greater than 0.5 or lesser than -0.5. Metabolites identified to have a VIP score greater than 1 through the OPLS-DA model between normal controls and patients with HF at stage C.

Supplementary Table S3. Significantly changed metabolites in patients with heart failure were selected in ESI negative mode.

| No. | Metabolites | m/z | RT  (min) | Fold change | | | P  (corr) | p-value  ANOVA | p-value  (N vs.C) | VIP  (N vs.C) |
| --- | --- | --- | --- | --- | --- | --- | --- | --- | --- | --- |
|  |  |  |  | **Log2**  **(A/N)** | **Log2**  **(B/N)** | **Log2**  **(C/N)** |  |  |  |  |
| Significantly increased metabolites in HF | | | | | | | | | | |
| 1 | LPE(18:0/0:0) (M-H) | 480.312 | 1.13 | 1.00 | 1.20 | 0.86 | -0.672 | 7.06E-10 | 2.70E-08 | 1.589 |
| 2 | Ceramide 24:0 (M-H) | 694.6404 | 13.66 | 1.06 | 0.97 | 1.46 | -0.653 | 2.50E-09 | 2.49E-08 | 2.685 |
| 3 | LysoPC(16:0) (M-H) | 540.3344 | 1.13 | 0.86 | 0.97 | 0.67 | -0.639 | 1.19E-08 | 1.67E-07 | 2.516 |
| 4 | Ceramide 22:0 (M-H) | 666.6086 | 13.13 | 0.81 | 0.74 | 1.01 | -0.572 | 4.69E-06 | 1.66E-06 | 1.573 |
| 5 | LysoPE(16:0) (M-H) | 452.2802 | 1.18 | 3.43 | 3.77 | 3.18 | -0.51 | 9.75E-09 | 2.39E-05 | 1.034 |
| 6 | Ceramide 24:0 (M-H) | 694.6404 | 13.3 | 1.13 | 1.10 | 4.15 | -0.509 | 1.19E-09 | 3.95E-05 | 1.011 |
| 7 | 2.26_834.5566 m/z | 834.5566 | 2.26 | > 6 | > 6 | > 6 | -0.504 | 2.58E-13 | infinity | 1.527 |
| 8 | 1.79_869.6034 m/z | 869.6034 | 1.79 | > 6 | > 6 | > 6 | -0.504 | 1.30E-07 | infinity | 1.453 |
| Significantly decreased metabolite in HF | | | | | | | | | | |
| 1 | PE(36:3) (M-H) | 740.5294 | 6.13 | -3.82 | -4.19 | -2.64 | 0.798 | 4.90E-30 | 1.22E-10 | 3.777 |
| 2 | PC(36:2) (M-H) | 830.5999 | 7.3 | -1.31 | -1.45 | -0.85 | 0.769 | 2.97E-22 | 1.25E-11 | 7.045 |
| 3 | PE(34:2) (M-H) | 714.513 | 5.97 | -2.76 | -2.88 | -2.20 | 0.766 | 2.07E-21 | 2.29E-09 | 3.975 |
| 4 | PC(34:2) (M-H) | 802.5757 | 5.56 | -1.30 | -1.27 | -0.78 | 0.718 | 6.48E-05 | 0.02336 | 11.962 |
| 5 | PC(36:3) (M-H) | 828.5828 | 5.71 | -3.12 | -3.42 | -1.32 | 0.712 | 1.14E-29 | 9.57E-10 | 2.908 |
| 6 | SM 24:1(M+FA-H) | 857.6831 | 11.92 | -1.21 | -0.98 | -1.56 | 0.64 | 3.37E-08 | 1.84E-07 | 1.998 |
| 7 | PE(34:1) (M-H) | 716.5291 | 7.38 | -1.00 | -0.84 | -0.64 | 0.636 | 7.69E-10 | 6.53E-07 | 4.009 |
| 8 | SM 24:2(M+FA-H) | 855.6673 | 9.00 | -1.71 | -1.88 | -1.06 | 0.633 | 2.14E-16 | 1.98E-07 | 3.642 |
| 9 | PE(36:4) (M-H) | 738.513 | 5.71 | -2.80 | -3.32 | -1.33 | 0.602 | 1.46E-19 | 4.69E-07 | 3.365 |
| 10 | PE(36:2) (M-H) | 742.5447 | 7.59 | -2.13 | -2.92 | -1.30 | 0.598 | 3.49E-14 | 2.87E-05 | 2.010 |
| 11 | PC(36:1) (M-H) | 832.6149 | 9.04 | -1.06 | -0.81 | -0.70 | 0.575 | 6.16E-10 | 6.56E-06 | 3.48 |
| 12 | PC(34:0) (M-H) | 806.5983 | 8.78 | -1.55 | -1.08 | -1.28 | 0.537 | 3.18E-08 | 8.27E-05 | 1.101 |
| 13 | LysoPE(20:4) (M-H) | 500.2809 | 0.94 | -2.31 | -1.82 | -1.21 | 0.529 | 2.95E-11 | 4.75E-05 | 1.638 |
| 14 | PE(36:1) (M-H) | 744.5601 | 9.71 | -1.72 | -1.46 | -1.44 | 0.515 | 1.72E-07 | 0.00027 | 1.416 |
| 15 | 5.55_870.5548 m/z | 870.5548 | 5.55 | -1.84 | -2.03 | -0.91 | 0.504 | 2.83E-12 | 0.00012 | 1.127 |

Metabolites of interest were extracted from S-plots constructed following OPLS-DA, and markers were chosen based on their p (corr) value greater than 0.5 or lesser than -0.5. Metabolites identified to have a VIP score greater than 1 through the OPLS-DA model between normal controls and patients with HF at stage C.
